# Editorial: Building climate resilient deciduous tree crops by deciphering winter dormancy

**DOI:** 10.3389/fpls.2024.1463405

**Published:** 2024-07-26

**Authors:** Shu Yu, Bénédicte Wenden, Louise Ferguson, Li Tian

**Affiliations:** ^1^ Department of Plant Sciences, University of California, Davis, Davis, CA, United States; ^2^ INRAE, Univ. Bordeaux, UMR Biologie du Fruit et Pathologie 1332, Villenave d’Ornon, France

**Keywords:** winter dormancy, tree crops, bud break, heat accumulation, climate change

Buds of deciduous tree crops enter winter dormancy in response to shorter photoperiods and lower temperatures in the fall. Winter dormancy comprises successive phases of endodormancy and ecodormancy. The transition from endodormancy to ecodormancy requires the exposure of dormant buds to a certain amount of chilling temperatures, whereas heat accumulation in ecodormant buds is essential for the successful shift from dormancy to active growth in tree crops (Lang et al., 1987). While bud dormancy allows plants to withstand harsh winters, timely bud break ensures their successful regrowth and production. As such, the initiation, progression, completion, and regulation of bud winter dormancy and break have been widely recognized for their importance in the survival and productivity of tree crops. However, climate change, often associated with warmer winters and temperature fluctuations (Inouye, 2022; Grossman, 2023), poses significant challenges to these processes, as alterations in temperature patterns can disrupt the delicate balance in temperature necessary for optimal bud dormancy and break.

This Research Topic centers on the biochemical, genetic, physiological, and environmental regulation of bud winter dormancy and its break in various woody perennials, with a focus on the impact of climate change. It also explores breeding strategies to adapt to climate change, particularly rising winter temperatures. The eight articles encompass studies conducted on three continents, covering a range of crops including nut, fruit, ornamental, and beverage crops. They are briefly highlighted below.

Gabay and Flaishman reviewed recent advancements in genetic and genomic resources as well as roles of genes, phytohormones, and metabolites related to dormancy regulation in pear (*Pyrus* spp.). They discussed the need for developing new pear cultivars with low chilling requirements and the challenges in pear breeding due to extended juvenility. They further emphasized the importance of understanding the genetic and physiological factors controlling bud dormancy in pear breeding. To this end, they pointed out the utility of quantitative trait loci (QTLs) in understanding dormancy regulation and developing genetic markers for marker-assisted selection in breeding. Lastly, the authors suggested Mediterranean climates could be used to simulate future temperate region climates for pear breeding.

Japanese plum (*Prunus salicina* Lindl.) trees require specific temperature conditions during bud dormancy for proper flowering in the spring. These requirements vary among cultivars due to genetic diversity (Guerra et al., 2009). Guerrero et al. analyzed the adaptation of 21 Japanese plum cultivars to future climate conditions using empirical data and climate projection models to predict chilling and heat accumulation (Delgado et al., 2021; Fadón et al., 2023). They found that the region of Badajoz in Spain may face challenges in meeting chilling requirements for dormant buds due to reduced winter chill, potentially limiting Japanese plum cultivation in these areas.

Scion/rootstock combinations are commonly used in pecan [*Carya illinoensis* (Wangenh.) K. Koch] production. Kaur et al. investigated the impact of low temperatures (-2, 0, 2, and 4°C) on bud break, leaf growth, and flower growth in different pecan scion/rootstock combinations. Their research showed that the low-temperature tolerance of these combinations correlated with their usage of bark soluble sugars and starches, as well as woody tissue soluble sugars. This insight is valuable for making informed decisions about scion/rootstock combinations in pecan orchards.

Leveraging integrated metabolite and transcriptional analysis, two studies explored biochemical and physiological mechanisms underlying bud dormancy release. Tang et al. dissected the regulatory network of bud sprouting in tea [*Camellia sinesis* (L.) O. Kuntze] by comparing metabolic and transcriptional profiles at different developmental stages. Their results highlighted the roles of plant hormones, glucose metabolism, and reactive oxygen species scavenging in the regulation of tea bud sprouting. In particular, soluble sugar reserves and oxidative stress before sprouting, and hormone regulation by zeatin during sprouting, led to bud dormancy release and active bud growth. Yu et al. used transcriptome analysis to examine gene expression profiles in pistachio (*Pistacia vera* L.) buds exposed to different winter chill accumulation at three orchard locations. Their study revealed increased expression of genes encoding enzymes breaking down callose and starch in endodormant buds, which facilitates endodormancy release and subsequent growth. Additionally, the decreased expression of genes involved in abscisic acid (ABA) biosynthesis suggested that lower ABA levels promote bud endodormancy release; this finding was supported by the observation of higher levels of carotenoid precursors and lower ABA content in buds undergoing endodormancy release.

To decipher the signaling mechanisms involved in bud dormancy establishment and release, Gai et al. applied a calcium (Ca^2+^) chelator and a Ca^2+^ channel blocker to dormant tree peony (*Paeonia suffruticosa* Andr.) buds, which resulted in delays in chilling- and gibberellic acid (GA)-induced endodormancy release. However, when Ca^2+^ was reapplied, the delay was alleviated, suggesting the involvement of Ca^2+^ in this process. Furthermore, the increased expression of several candidate calcium sensor genes following chilling and GA treatment suggests a potential role for calcium in bud endodormancy release, although further investigation is needed to determine if calcium functions as a signaling molecule or a nutrient in this process.

To identify genes participating in the bud break process, Mao et al. focused their study on *PsATL33*, a gene encoding a RING-H2 finger protein. They previously discovered that *PsATL33* exhibited differential expression in chilling-treated tree peony buds (Yuan et al., 2024). In this study, overexpression of *PsATL33* in petunia (*Petunia hybrida* ‘Mitchell Diploid’) accelerated seed germination, increased leaf size, and promoted axillary bud growth. Conversely, transiently silencing of *PsATL33* in tree peony delayed bud break and growth, suggesting its role in releasing seeds and buds from dormancy. Additionally, this research revealed a connection between *PsATL33* expression and the production of bioactive GA. The investigation by Watson et al. employed targeted capture sequencing on apple (*Malus domestica* Borkh.) to identify single nucleotide polymorphism (SNP) markers for dormancy- and flowering-related genes, as well as those in the interval of a previously identified QTL implicated in bud break. Through Genome-wide association studies (GWAS) analysis of 239 apple cultivars using these SNP markers, a SNP in *MdPRX10*, a candidate peroxidase gene, was associated with late bud break. The authors observed that the expression pattern of *MdPRX10* was modulated by exposure to chilling temperatures. They proposed that *Md*PRX10 integrates temperature cues in dormancy pathways through redox-mediated signaling and regulation of C-repeat binding factor (CBF) genes associated with cold tolerance.

Collectively, these studies have enhanced our understanding of the genetic, physiological, and environmental factors that regulate bud winter dormancy and break in tree crops. They have also provided valuable insights for breeding programs, orchard management, and climate adaptation strategies. By comparing dormancy break mechanisms among different fruit and nut species, both conserved and species-specific genes and pathways could be identified. Additionally, future investigations could harness advanced breeding methods such as genomic selection to improve breeding efficiency by predicting and selecting desirable traits associated with bud winter dormancy and break.

## Author contributions

SY: Writing – review & editing. BW: Writing – review & editing. LF: Writing – review & editing. LT: Writing – review & editing, Writing – original draft.
